# How to achieve the desired outcomes of advance care planning in nursing homes: a theory of change

**DOI:** 10.1186/s12877-018-0723-5

**Published:** 2018-02-14

**Authors:** J. Gilissen, L. Pivodic, C. Gastmans, R. Vander Stichele, L. Deliens, E. Breuer, L. Van den Block

**Affiliations:** 1 End-of-life Care Research Group, Vrije Universiteit Brussel (VUB) & Ghent University, Laarbeeklaan 103, 1090 Brussels, Belgium; 2 Centre for Biomedical Ethics and Law, KU Leuven, Kapucijnenvoer 35, Box 7001, 3000 Leuven, Belgium; 3 Department of Pharmacology, Ghent University, De Pintelaan 185, 9000 Ghent, Belgium; 4 Department of Medical Oncology, Ghent University Hospital, De Pintelaan 185, 9000 Ghent, Belgium; 5 Alan J Flisher Centre for Public Mental Health, Department of Psychiatry and Mental Health, University of Cape Town, Cape Town, South Africa; 6 Department of Family Medicine and Chronic Care, Vrije Universiteit Brussel (VUB), Laarbeeklaan 103, 1090 Brussels, Belgium

**Keywords:** Advance care planning, Complex intervention, Implementation, Intervention development, Medical Research Council framework, Nursing home, Theory of change

## Abstract

**Background:**

Advance care planning (ACP) has been identified as particularly relevant for nursing home residents, but it remains unclear how or under what circumstances ACP works and can best be implemented in such settings. We aimed to develop a theory that outlines the hypothetical causal pathway of ACP in nursing homes, i.e. what changes are expected, by means of which processes and under what circumstances.

**Methods:**

The Theory of Change approach is a participatory method of programme design and evaluation whose underlying intention is to improve understanding of how and why a programme works. It results in a Theory of Change map that visually represents how, why and under what circumstances ACP is expected to work in nursing home settings in Belgium. Using this approach, we integrated the results of two workshops with stakeholders (*n* = 27) with the results of a contextual analysis and a systematic literature review.

**Results:**

We identified two long-term outcomes that ACP can achieve: to improve the correspondence between residents’ wishes and the care/treatment they receive and to make sure residents and their family feel involved in planning their future care and are confident their care will be according to their wishes. Besides willingness on the part of nursing home management to implement ACP and act accordingly, other necessary preconditions are identified and put in chronological order. These preconditions serve as precursors to, or requirements for, accomplishing successful ACP. Nine original key intervention components with specific rationales are identified at several levels (resident/family, staff or nursing home) to target the preconditions: selection of a trainer, ensuring engagement by management, training ACP reference persons, in-service education for healthcare staff, information for staff, general practitioners, residents and their family, ACP conversations and documentation, regular reflection sessions, multidisciplinary meetings, and formal monitoring.

**Conclusions:**

The Theory of Change map presented here illustrates a theory of how ACP is expected to work in order to achieve its desired long-term outcomes while highlighting organisational factors that potentially facilitate the implementation and sustainability of ACP. We provide the first comprehensive rationale of how ACP is expected to work in nursing homes, something that has been called for repeatedly.

## Background

Advance care planning (ACP) is a process that supports adults at any age or stage of health in understanding and sharing their personal values, life goals, and preferences regarding future (medical) care [1, 2]. If a person chooses so, the contents of such conversations can be set down in writing [3, 4]. ACP is of particular relevance for frail older adults, considering their unpredictable and prolonged dying trajectories characterised by multiple cognitive and functional limitations [5–7]. Despite the sizeable portion of older people who remain at home until death [8, 9], circumstances sometimes require them to move to a nursing home [5, 10, 11]. In Belgium in 2013, 11% of people aged 75 and over and 26% of people aged 85 and over lived in a long-term care facility such as a nursing home [12]. This makes the nursing home a particularly relevant setting for ACP.

However, the actual implementation of ACP in nursing home practice seems to be a worldwide challenge. Recent studies have shown that there is still a low prevalence of ACP engagement among older adults [13–15] and that fewer than 11% of nursing home residents in Germany (2012) have completed an advance directive [16]. This is also the case in Flanders, Belgium. Although ACP policy documents are available in 95.1% of Flemish nursing homes [17–19] and orders from general practitioners (GP orders) are relatively common among Flemish nursing home residents with dementia (59%), only 3% has an advance patient directive and 8% has assigned a legal representative at time of death [20, 21].

ACP is a complex intervention with multiple components operating at different levels of the healthcare system [22], and until now it has been unclear what the effective elements of the intervention are and how or in what circumstances ACP can best be implemented in routine nursing home care [15, 23–25]. To provide a more detailed understanding of the effective elements and such circumstances, frameworks such as those from the Medical Research Council (MRC), the TIDieR checklist for better reporting of interventions, the MORECare statement or the multiphase optimization strategy (MOST) state that prior to modelling and evaluating an intervention, those developing them should specify the processes through which and the circumstances under which the intervention is expected to lead to the desired change [22, 26–30]. The MRC further articulates the importance of ‘theory’ and states that researchers should develop or report the logic model or theory behind the intervention early on, “to focus on the most important uncertainties that need to be addressed and hence advance understanding of the implementation and functioning of the intervention” [22]. While there is literature outlining how interventions are supposed to be delivered, only a few reported their development, including the outline of an a-priori rationale, logic model or theory. It has been suggested that ACP can be informed by health behaviour models [31, 32] such as the Representational Approach to Patient Education, as described in a recent study from Song and Ward (2015) [33]. However, except for the latter example, we have found no description of the development or use of such theory to inform intervention development for or evaluation of a comprehensive ACP programme in the nursing home setting. This is in fact a common problem identified in non-pharmacological (e.g. psychosocial and educational) intervention studies in general [34].

## Aim

In this study, we aimed to develop a theory that outlines the hypothetical causal pathway of ACP in nursing homes, i.e. which changes are expected and how, through which processes and under what circumstances. This serves as a first step in the development of an ACP intervention for the nursing home setting.

## Methods

### Design

A Theory of Change approach was used to develop a ‘theory of change’ for ACP using input from stakeholders from various backgrounds in two workshops. We integrated the results of these workshops with the results of a contextual analysis, a systematic literature review about preconditions for successful ACP in nursing homes (published elsewhere [35]), and relevant literature in the field.

### Theory of Change approach

Following the Aspen Institute and Centre for Theory of Change, a Theory of Change (ToC) is “a theory of how and why an initiative works which can be empirically tested by measuring indicators for every expected step on the hypothesised causal pathway to impact” [36]. This is visualised in a ‘ToC map’, which provides a comprehensive illustration of how long-term outcomes can be achieved in a specific context and under particular circumstances [36, 37]. Within this map specific terms are used (see Table 1).

**Table 1:** Theory of Change terminology

Terminology	Definition (adapted from De Silva, 2015 [36])
Impact	The real-world change we are trying to achieve in nursing homes.
Ceiling of accountability	The point at which we stop accepting responsibility for achieving those outcomes solely through the intervention programme.
Long-term outcomes	The outcome that the programme is able to achieve on its own. This can inspire the choice for particular primary and secondary outcomes in the evaluation of the intervention.
Preconditions	A precondition or intermediate outcome is a necessary requirement, condition or element that needs to be realized for the desired outcome to be achieved. In the context of ACP, these preconditions are the precursors or requirements for accomplishing successful ACP.
Intervention	The different components of the complex intervention. They represent certain “actions” that need to be undertaken to bring about a certain result, intermediate outcome or precondition. These are “those things that the programme must do to bring about the outcomes”.
Assumptions	An external condition beyond the control of the project that must or is assumed to exist for the outcome to be achieved.
Rationales	The facts or reasons (based on evidence or experience) behind the choice of the intervention activities or strategies and each link of the causal pathway.

ACP advance care planning

The process used to create a ToC map is “backwards outcome mapping”. This means that one starts by defining the ultimate impact and long-term outcomes that are to be achieved. From this point, working backwards means that all preceding intermediate outcomes or “preconditions” required to reach this envisioned impact are defined. Because this is different to the conventional “so-that” reasoning, as it is called, it allows better reflection on the reality of how this intervention will achieve impact [38].

In this paper, we illustrate the process of developing a ToC map as part of the development phase of an ACP intervention. It is suggested by De Silva et al. (2014) that it has the potential to strengthen the MRC framework in all four of its phases: I) development, II) feasibility/piloting, III) evaluation and IV) implementation. During development, a ToC approach may enhance stakeholder engagement, improve the initial design of the intervention and help tailor the intervention to its specific context. During feasibility and pilot testing, it can highlight barriers to implementation and test the acceptability and applicability of the intervention in more detail. In the evaluation phase, the ToC map can enable a comprehensive evaluation of the implementation process to disentangle the key features of its effectiveness [36]. Combining the experience of implementation and evidence gathered in the evaluation phase, this map can subsequently be revised to produce a ‘story’ of how ACP worked in a particular setting [36].

### Setting

We performed this study in Flanders, where 60% of the Belgian population lives (approximately 6.5 million people out of a total of 11 million). Flemish nursing homes are facilities providing skilled nursing care for older adults who have problems with daily life activities and/or cognitive capacity. Medical care, including end-of-life care, is usually provided by external general practitioners (GPs) who are not part of the regular team of professionals in the nursing home [39]. However, nursing homes are legally obliged to have at least one coordinating and advisory physician (CAP) (remunerated according to the number of beds), who coordinates medical care in the facility, as well as reference nurses for palliative care (0.10 FTE per 30 residents) [40, 41]. Together they are responsible for embedding a “palliative care culture”, sensitising staff about palliative care, providing GPs with advice, and organising specific training on palliative care [35]. However, the training and accreditation of these physicians and nurses in palliative care is not legally regulated, which makes it unclear to what extent they can actually impact daily practice.

### Steps to develop the Theory of Change map

We undertook six steps to develop the ToC map: 1) contextual analysis, 2) systematic literature review, 3) first ToC workshop with stakeholders, 4) meetings with core research team, 5) second ToC workshop with stakeholders and 6) finalizing meetings with core research team. Table 2 outlines the goals, methods and output of each of these steps. The results of the systematic review (step 2) are published elsewhere [35]. In the following section, we describe in more detail which stakeholders were selected to take part in the workshops and how these were structured to develop the ToC map.

**Table 2:** Aim, methods and output of each step in developing Theory of Change map

Step	Aim	Methods	Output
1|	To obtain full background information on ACP in Flanders and the nursing home context	Contextual analysis by means of: (literature) review of existing policies, national guidelines, national studies of ACP in the Flemish nursing home setting (e.g. EU FP7 project 'PACE') and local/national ACP initiatives for the nursing home setting	Background report listing possible barriers and facilitating factors^a^ for ACP in nursing homes related to 1) the resident (e.g. average time of stay in a nursing home is 3 years), 2) family (e.g. family listed as contact person often not according to regulated cascade system^b^), 3) involved care professionals (e.g. GPs in Flanders are not employed by nursing home facilities), 4) facility (e.g. staff shortages), 5) Belgian/Flemish (healthcare) system (e.g. ACP policy not driven by law; existence of formal quality indicators)
2 |	To identify the preconditions related to successful ACP in the nursing home setting	Systematic review* of empirical studies and reviews (2005–2015) about ACP in nursing homes, by the core research team	List of preconditions for ACP in the nursing home setting to be used during workshop 1 to trigger discussion
3 |	To create a first draft of the ToC map	ToC stakeholder workshop 1 by ToC facilitators (LVDB and LP) and stakeholders	First draft of ToC map, including: ▪ Impact, ceiling of accountability and long-term outcomes ▪ Preconditions/intermediate outcomes, including their chronological order ▪ List of possible interventions, assumptions and rationales
4 |	To create a second draft of the ToC map based on integration of output from steps 1, 2 and 3	Several meetings with core research team to construct a draft ToC map	Second draft of ToC map, including: ▪ Reformulated impact and long-term outcomes ▪ Preconditions chronologically ordered and coloured according to level to which they are applicable ▪ Precondition “support by an external trainer” (suggested by research team) ▪ Possible interventions (added by the research team) such as the availability of a trainer and a monitoring system
5 |	To refine the second draft ToC map, to fill in the gaps and to get consensus on the chronological order of the hypothesised causal pathway	ToC stakeholder workshop 2 by ToC facilitators and stakeholders in which second draft of ToC map (output of step 4) is presented	Refined draft of second ToC map, including: • Redefined secondary outcome to be measurable ▪ Additional elements, added in step 4, approved by stakeholders ▪ Details added by stakeholders (e.g. which healthcare professional is responsible for implementing ACP, re-named ACP facilitator as “ACP reference person”) ▪ Additional arrows added by stakeholders
6 |	To develop the final draft ToC map that outlines the hypothetical causal pathway of ACP in nursing homes based on integration of output from steps 1 to 5	Several meetings with core research group to construct the ToC map, review by a ToC expert, comparison with existing ToC maps from other research projects and consultation of implementation science literature (in general and about ACP) and relevant theoretical models	Further integration of outputs of steps 1–5 into a final draft of a ToC map (presented in Fig. 1) and narrative, including: ▪ Preconditions merged or reformulated and put in chronological order ▪ Numbers added to mark interventions ▪ Rationales and assumptions written up by the core research team in a separate document (narrative), based on stakeholders’ and researchers’ experience, literature and relevant theoretical models

*ToC* Theory of Change, *ACP* advance care planning, *GP* general gractitioners

*The results of this systematic review are elsewhere [35]

^a^Barriers are defined as contextual elements that inhibit ACP in Flemish nursing homes; Facilitators are defined as contextual elements that can support ACP in nursing homes

^b^A hierarchical system that regulates who functions as the legal representative/surrogate decision-maker if the person/patient has not assigned a legal representative him−/herself and lacks the mental capacity to make the decisions that have to be made: 1) the spouse or (legal) cohabiting partner, 2) an adult child of the patient, 3) a parent, 4) an adult sibling of the patient, 5) the professional carer representing the patient’s interests in multidisciplinary consultations

#### Theory of Change stakeholder workshops

We organised two half-day ToC workshops with stakeholders (June 29^th^ and July 13^th^ 2015) following the methodology outlined in the available ToC manuals [42, 43].

#### Stakeholders

Stakeholders were defined as people involved in the development, implementation or organisation of ACP in nursing homes. We purposively sampled and recruited stakeholders using a variety of criteria including: (i) affiliated with a Flemish nursing home OR having knowledge of the Flemish nursing home setting OR whose work in policymaking or research influences care in Flemish nursing homes; AND (ii) being acquainted with ACP through their work. All stakeholders were recruited by JG by means of e-mails and follow-up telephone calls, through contacts that were established in previous work regarding ACP and through the research group’s network of experts in ACP practice. We sent out 30 invitations to potential stakeholders and 21 of those people participated. The stakeholders who attended the two workshops were not always the same people, but a key group of stakeholders (*n* = 6) attended both to ensure continuity between the two workshops. Characteristics of the participating stakeholders can be found in Table 3.

**Table 3:** Characteristics of stakeholders in the Theory of Change workshops (*n* = 2)

Characteristics	Workshop 1 (*n* = 12)	Workshop 2 (*n* = 15)^b^
Gender		
Male	1	4
Female	11	11
Primary profession		
Care professional		
General practitioner (GP)	1	1
Coordinating and advisory physician (CAP)	0	1
Nurse (including public health nurses)	2	2
Palliative care reference nurse	1	2
Psychologist (one of whom is involved in research linked to ACP)	2	2
Social worker	1	0
Physiotherapist	1	1
Dementia reference person	0	1
Other		
Nursing home management	2	2
Ethicist	1	1
Health sociologist	0	1
Representative of council for the elderly	1	1
Employer^a^		
Nursing home	7	7
Private practice	1	0
University	3	3
Overarching organisation	1	1
National council for the elderly	1	1

^a^Multiple options are possible

^b^The total number of unique participants was 21. Six participants attended both the first and the second workshop (1 nurse, 1 palliative care reference nurse, 2 psychologists, 1 social worker, 1 nursing home manager)

#### Procedure

Each workshop was structured to include a brief introduction of the project and the ToC approach, the importance of ACP in nursing homes and a mapping exercise using structured group discussions and small group exercises. These ToC workshops are characterised by their output, a ToC map (and gaining agreement on this among the involved stakeholders) rather than just giving views and opinions. In addition, the ToC facilitators generally have a more active role than those moderating focus groups, given that the aim was not only to obtain participants’ views but to create a ToC map together. Table 4 shows the central themes and questions asked in each workshop.

**Table 4:** Central themes and questions asked in the Theory of Change stakeholder workshops

Workshop 1 and 2
a) Problem description b) Introduction to ToC method and ground rules (e.g. “Everyone’s input is equally valid”, “Think outside the box”, “Give the facilitator time to write things down”, “Nothing that is written down is definitive. We are following an iterative process”) c) The question to initiate reflection: “In an ideal world, what would need to happen for a successful implementation of ACP?”
Workshop 1
a) Agreement on impact: What is the fundamental change we want to see in the nursing home setting in Flanders? How will the Flemish nursing home community be different because of what we do? b) Ceiling of accountability c) The long-term outcomes of advance care planning in nursing homes d) What are the intermediate preconditions that are necessary to produce the long-term outcomes? Why do we think a given precondition will lead to (or is necessary to) reach the one that follows it? e) What contextual conditions or circumstances are necessary to achieve the preconditions? f) Consensus concerning the chronological order of preconditions
Workshop 2
a) Presentation and discussion of the ToC map developed in workshop 1 b) Review and refinement of the ToC developed in workshop 1 and filling in the gaps: Is the ToC map presented here “feasible” (likely to work), “effective” and “sustainable”? Is the change logically displayed? Are there essential elements that are missing or that we should definitely consider or discuss? c) Which interventions should be initiated to achieve the preconditions and the long-term outcome?

*ToC* Theory of Change, *ACP* advance care planning

LVDB and LP, trained in the use of ToC, facilitated both workshops. The results of the context analysis (step 1) and the systematic review (step 2) [35] were used to provoke discussion and prompt questions concerning the preconditions found most important in the literature to achieve the long-term outcome and to check whether all levels of change (the individual level (resident or family); the professional level (GP or nursing staff) and the facility level (nursing home)) were considered.

During the first ToC workshop (step 3), the impact and long-term outcome of ACP in nursing homes was defined, after which participants worked 'backwards' to map all preconditions, using visual aids (post-its on a whiteboard). This process was repeated iteratively until consensus about the content and chronological position of the preconditions was reached. After this workshop, JG drafted a ToC map, which was then discussed during two meetings with the core research team to review the outcomes of the ToC workshops and the draft of the ToC map (step 4). The aim of the second workshop (step 5) was to reach consensus among all stakeholders about the preconditions, their positioning in the ToC map and to formulate intervention components and activities needed to attain the preconditions. The facilitators presented the draft ToC map created in step 4 in poster format, to make sure all participants shared a similar understanding of the causal pathway presented in the map. At both workshops, the participants were encouraged to reflect on their reasoning or rationales of how and why certain preconditions lead to the next, why certain interventions are necessary for desired outcomes to be achieved and to make explicit their assumptions about possible implementation barriers in the local context.

After the second workshop, the core research team met four times (step 6) to discuss the formulation of the preconditions, their potential causal relationship, and the intervention components in the ToC map. During this step, a ToC expert (EB) reviewed the methods and terms used to ensure they were used correctly and to check the consistency of the causal pathway. The map was subsequently checked against relevant literature proposed by the core research group and the four attributes (plausible, doable, meaningful and testable) for a good theory of change [43].

### Data analysis

The first author transcribed video and audio recordings of the workshops (to which participants gave verbal consent) and took photographs of the ToC map at the end of each workshop to maintain a visual record. Points that were raised and perceived as important by the majority of stakeholders were included in the map. The first author constructed the ToC maps using Lucidchart (http://www.lucidchart.com).

## Results

**Fig. 1:**
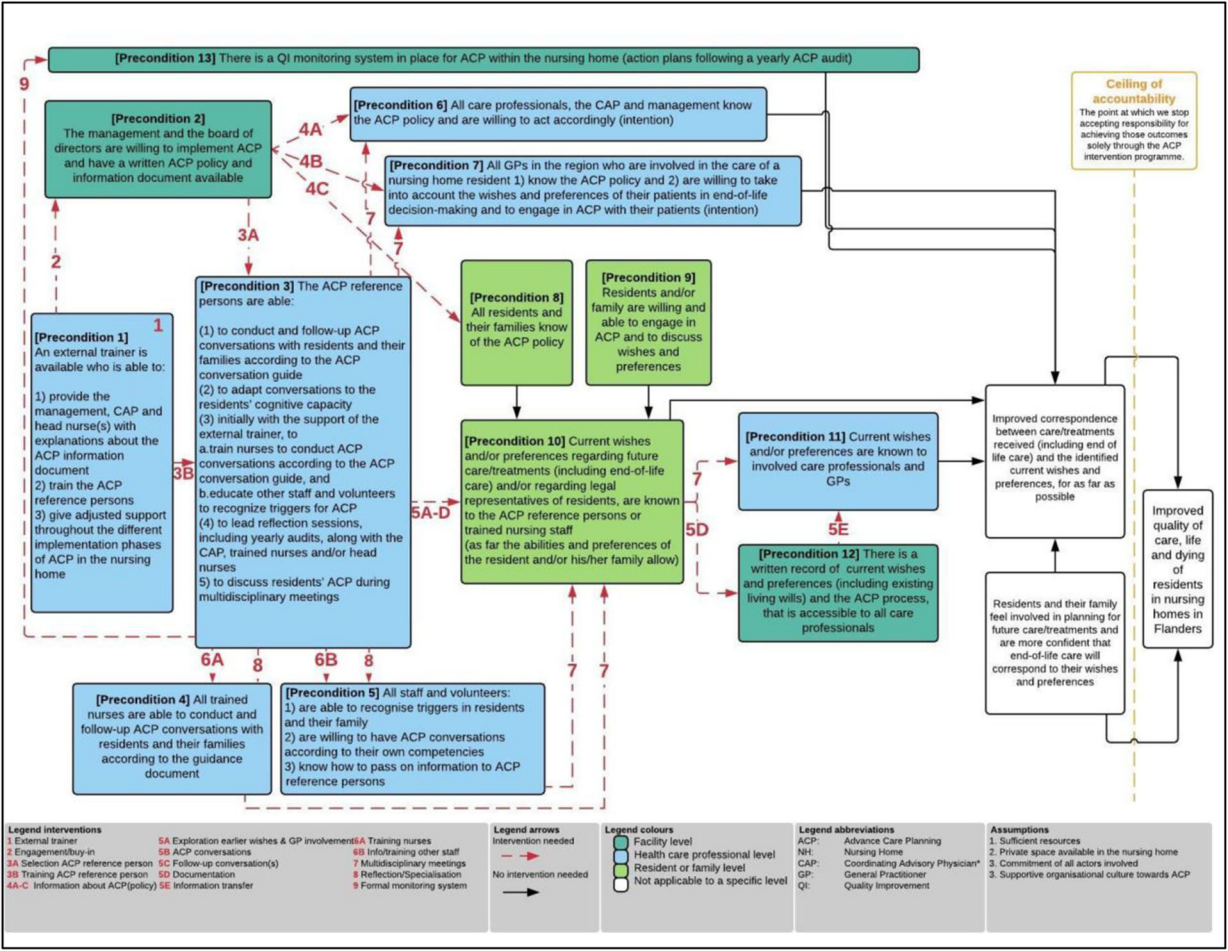
Theory of Change map. ACP advance care planning; QI quality improvement; CAP coordinating advisory physician; GP general practitioner. *Since 2000, each nursing home is legally bound to have a coordinating advisory physician (CAP), a general practitioner, preferably trained in gerontology, whose tasks include some of those related to individual end-of-life care situations (consultancy, taking charge of care, or conflict mediation) [81, 82]

As suggested in the Checklist for Reporting Theory of Change, we present i) impact, ii) ceiling of accountability, iii) long-term outcomes, iv) preconditions, v) interventions and vi) assumptions [44]. These should be read in conjunction with the ToC map presented in Fig. 1.

### Impact

The desired ultimate impact that should be achieved in nursing homes was identified as “improved quality of care, quality of life and quality of dying in nursing homes in Flanders”.

### Ceiling of accountability

The threshold at which the ACP intervention is no longer directly accountable for the desired impact is delineated by the ‘ceiling of accountability’, which is situated between the impact ‘improving quality of care, life and dying’ and the long-term outcomes. ACP cannot achieve the formulated impact solely on its own (e.g. other personal factors and factors pertaining to the healthcare organisation, healthcare system, and the broader environment may also affect the quality of care of someone in the nursing home) though ACP may contribute to achieving the impact through its effect on the long-term outcomes, that are described below.

### Long-term outcomes

We identified two long-term outcomes that are desired to be achieved by ACP:

1) “Correspondence between the care/treatments received (including end-of-life care) and the current wishes and preferences identified, as far as possible”. Care and/or treatments received do not always align with care/treatments preferred. However, a correspondence between the two is identified as the most important outcome for assessing the effects of ACP in nursing homes, and critical to improve care, quality of life and quality of dying [45]. It is also reported as the primary or secondary outcome in a wide array of effectiveness studies [15, 23, 46–48] and as a primary objective of ACP and in ACP definitions [25, 45, 49, 50].

2) “Residents and/or their family feel involved in planning future care/treatments and are more confident that end-of-life care will correspond to their wishes and preferences”. Residents and families appreciate feeling prepared for the future and want their wishes and preferences regarding care and treatment to be considered seriously by the healthcare professionals involved [51, 52].

### Preconditions

Based on the results of the systematic review [35] and ToC workshops, we identified 13 important preconditions that need to be fulfilled for the desired long-term outcomes to be achieved. All preconditions are presented in the coloured boxes in Fig. 1, which should be read from left to right. The distinct colours indicate the level to which each precondition is most applicable. Most preconditions are applicable to healthcare professionals within the nursing home.

The ToC map, as shown in Fig. 1, first identifies the availability of a sufficiently skilled trainer [precondition 1], who is available for all participating nursing homes, as an essential first step in the implementation of an ACP intervention. Next to this trainer, who is external to the organisation, the engagement of the nursing home management is necessary [2] to ensure full integration into routine nursing home care provided by in-house staff, therefore this includes assigning staff that function as ‘ACP reference persons’ [3]; trained nurses that are able to conduct ACP conversations [4]; trained staff that is able to signal triggers for ACP and knows how to pass on this information [5]; informed care professionals [6], GPs [7] and residents and their families [8, 9]; and care professionals that have the intention to take into account the wishes and preferences of nursing home residents and all to be willing to engage in ACP. That wishes and preferences are known to the ACP reference persons or trained facilitators (through ACP conversations) is a key outcome in the ToC map [10]. This is followed by the need for all involved care professionals to know these wishes [11] and the availability of a written record that is accessible [12]. To ensure quality of ACP is held high-standard, ongoing monitoring is necessary [13]. If all the preconditions described in the ToC map are achieved, nursing home residents who engaged in the ACP programme and their families should feel more involved in planning for the future and should feel confident that care will correspond to their preferences, for them to eventually have improved correspondence between the care/treatment they are actually receiving and those wishes and preferences.

### Interventions

Nine intervention components are required to fulfil each precondition. These are marked in Fig. 1 with dotted red arrows and numbers. In this section, we describe these interventions and their rationales in more detail.

1. Selection of external ACP trainer responsible for helping with gradual implementation of the intervention.

To carry out the tasks required in precondition 1, the stakeholders all agreed that an appropriately skilled external ACP trainer should be appointed to provide information, training and support, i.e. someone responsible for helping the staff throughout this change process of gradually implementing ACP into routine nursing home care. The intensity of the trainers’ support should gradually decline as implementation progresses and the nursing homes and their healthcare staff become more skilled in organising and structuring ACP themselves.

Studies and models of change show that people and organisations progress through a series of stages or phases when modifying behaviour or organisational structures with the help of interventions [53–55]. Such stages usually contain a preparation phase, an action phase or implementation phase and a maintenance or consolidation phase. Therefore, all intervention components and activities should be implemented gradually in a step-by-step approach.

2. Ensuring engagement and buy-in by the nursing home management.

To make sure the management and Board of Directors are willing to implement ACP (precondition 2), the external trainer has one or more meetings with them to establish their engagement and ensure buy-in into the project. The trainer also assesses the extent to which an ACP policy is already available within the nursing home and how it can be combined with the intervention and the ACP guidance document, which is part of the intervention. This guidance document provides detailed information about what ACP is, when and how it works and how ACP processes should be structured. The document is based on existing guidelines available in Belgium and internationally [56, 57].

Ensuring management commitment is important in processes that aim to effect change in current practice [55, 58]. Research has shown that management support ensures that all staff has a good understanding of how to use the programme effectively and appropriately, with the result that it is more likely to be sustained [58, 59]. An institutional policy or guideline is shown to support the process of ACP and to promote its implementation [60].

3. Selection and training of ACP reference persons.

ACP facilitators or “ACP reference persons” (healthcare professionals employed by the nursing home) should be appointed (3A) and receive training (3B) in order to have the skills necessary to accomplish the tasks highlighted in precondition 3, i.e. conducting conversations, training other staff, organising reflection sessions, performing monitoring and organising multidisciplinary meetings. These reference persons should market the programme, communicate the high priority of ACP for nursing home residents, provide education to other nurses, healthcare staff and volunteers, and perform regular monitoring to audit ACP processes and outcomes within the nursing home. The ACP reference persons are the main persons responsible for ensuring ACP is implemented in the home (with the support of the external trainer) and for performing scheduled and manualised ACP conversations. They are chosen in consultation with the management of the nursing home. The management and reference persons subsequently identify an additional number of nurses (or other paramedic staff) who are also competent to do ACP conversations. Both ACP reference persons *and* a limited number of such carefully selected nurses (or other paramedic profiles) were identified in the workshops as responsible for performing scheduled and manualised ACP conversations, to increase feasibility (i.e. decrease work load per person) and sustainability. The ACP reference persons need somewhat different skills to the external ACP trainer, because the latter is mainly responsible for supporting the ACP reference persons by providing them with the necessary tools and training to gradually implement ACP and optimize the change process in their facility (e.g. resistance, coordination, providing a structure). The ACP trainer’s support is intensive at the beginning of implementation, but decreases throughout the process as the ACP reference persons become increasingly more autonomous.

Reference persons are identified as a successful factor in much implementation science literature and healthcare research [59, 61, 62]. The reference persons are appointed among the professionals employed by the institution because evidence suggests that the use of ‘external’ facilitators does not enhance the sustainability of ACP, since they leave once the implementation period is over [60].

4. Information about ACP for staff, GPs, residents and their families.

To achieve preconditions 6, 7 and 8, all care professionals, the CAP, management (4A), the GPs involved (4B) and the residents and their families (4C) should be informed about ACP and the ACP policy within the nursing home using brochures, letters, information sessions or resident/family councils.

Lack of knowledge of ACP has been shown to be a barrier to engage in or successfully implement ACP [35]. Being fully informed about ACP helps people to accept why it is needed, be adequately prepared, make effective decisions, counter reluctance from both professionals and residents or families, and for residents to be able to share their care preferences adequately [35, 58].

5. ACP conversations and ACP documentation.

Precondition 10 requires the current wishes and preferences of the resident to be known. A guidance document based on existing guidelines [56, 57] is made available, outlining how conversations and documentation should be organised. After the resident is informed about the existence of the ACP policy in the nursing home and before they are invited for an initial ACP conversation, the ACP reference person or trained professionals (see intervention 6) explore whether the resident’s wishes and preferences have been documented in the past and how the residents’ GP wants to be involved in his/her patient’s ACP process (5A). At least two months after admission and following an evaluation of mental capacity, every resident, who is able to participate and/or family members who are found to be significant (or their legal representative), are invited to participate in the first conversation (5B). Several follow-up ACP conversations are organised: when circumstances change, if nursing home staff signal any important triggers, and annually (5C). Outcomes of conversations are always documented (5D) in written records in the residents’ files, where they are easily accessible to other care providers. In the event of a transfer to another care setting, the relevant information from the written record should accompany the resident (5E).

Regular follow-up is important as wishes and preferences can change with time, particularly if circumstances are different [45]. For example, this could happen when the resident’s health status changes (e.g. sudden deterioration or an additional diagnosis) or after a transition between hospital and the nursing home. Moreover, decisions take time and cannot be completed in one conversation [63]. Documenting residents’ preferences increases the likelihood that their wishes will be followed [23]. In addition, to ensure that care is provided as preferred, these preferences must be clearly documented in a written format and must be rapidly accessible when clinically relevant [35].

6. In-service training to nursing home staff and volunteers.

Two specific interventions are required to make sure that, besides the ACP reference persons, other nurses (or paramedic staff, as decided by the nursing home) are also able to conduct and follow up manualised ACP conversations (precondition 4), and that all other nursing home staff are involved and able to recognise meaningful triggers that signal that the resident or family wants to, is ready for or has a need to engage in an ACP conversation (precondition 5). Nurses receive regular in-service training about ACP conversations (6A). In addition, other nursing home staff (regardless of their age and specialism, including activity leaders, volunteers, night personnel, etc.) receive regular in-service training to help them recognise and signal triggers (6B). The training sessions for the latter will focus on signalling triggers for ACP and engaging in spontaneous conversations about related topics, hence differ from those for staff performing manualised ACP conversations according to the guidance document. Both types of trainings should be organised regularly by the appointed ACP reference persons.

In-service staff education is shown to be essential to enable implementation and ensure that the programme remains an effective part of standard care, even after an external trainer’s engagement period has ended [58, 59, 61, 64]. Nursing home residents usually have complex health trajectories where pending death and other triggers for ACP are not always recognised by the staff, who are often not trained in palliative care or similar areas [56, 65]. Because it is also important for residents and families to be able to have spontaneous ACP conversations as well as the ones that are scheduled, it is the responsibility of all professionals in the institution, including the hairdresser, to be able to engage in spontaneous conversations about such topics, according to their own competencies and within the bounds of their profession. For example, the resident may bring up the subject of future care and treatment while visiting the hairdresser [64]. Finally, these training sessions should happen regularly, as staff turnover can be high [66].

7. Multidisciplinary meetings.

To ensure the current wishes and preferences of the residents are known to all care professionals and GPs, as required in precondition 11, ACP conversations held with residents or their representatives and changes to ACP documentation should be regularly discussed in multidisciplinary meetings.

The importance of teamwork to achieve goals is supported by theories related to team effectiveness [55], scientific literature [58, 64] as well as the practical experience of the stakeholders.

8. Regular reflection sessions.

To ensure nurses, care professionals and volunteers learn from, support and communicate with each other, the ACP reference persons facilitate regular reflective sessions held among nursing home staff, for example using significant event analysis, which enables staff to reflect on ACP and analyse significant events with the aim of improving ACP practice where possible.

Reflective debriefing is shown to help staff feel supported and valued, and enhance their ability to teach each other and to develop understanding and critical thinking [67]. According to the stakeholders, these sessions can also function as ‘post-training’ support.

9. Formal monitoring, including audit, feedback and action plans.

To ensure that long-term outcomes of ACP are achieved and high-quality ACP is provided, a formal monitoring system is put in place. A system of this kind is an assessment of practice to know if efforts to change are working or additional efforts are needed. It should integrate audit, feedback and, if necessary, action plans to improve practice and enable quality improvement [68].

To ensure that all care professionals adhere to residents’ preferences, real-time monitoring through auditing and formal feedback on performance to the healthcare professionals involved are considered to be key drivers in implementing and sustaining new programmes [59].

### Assumptions

Assumptions are defined as the contextual conditions that need to be in place for ACP to function successfully. A failure to provide these creates barriers that may hinder the achievement of the long-term outcomes. Based on the results of the systematic review [35], stakeholders’ views and the contextual analysis, we identified the need for: sufficient resources (including funding, time and human capacity); a quiet private space where ACP conversations can be held; the commitment of everyone involved; a culture supportive of ACP in the nursing home so people feel free to reflect on and talk about death, dying and end-of-life issues; and an organisational culture that stimulates professionals to invest in ACP, despite the lack of financial incentives, staff shortages or staff turnover.

## Discussion

Using the Theory of Change approach, we have developed a theoretical framework for ACP in nursing homes that makes explicit what changes are expected as a result of ACP, how change can be achieved in long-term outcomes in nursing homes and under what circumstances. This is presented in a structured and logical ‘ToC map’. This ToC map provides a summary of ACP as a complex intervention and makes explicit the hypothesised causal pathway through which all intervention components of ACP interact to achieve the intended long-term outcomes: 1) improved correspondence between care/treatments received and current wishes and preferences, and 2) residents and family feeling more involved and confident that end-of-life care will correspond to their wishes. By achieving these long-term outcomes, we aim to improve the quality of care, quality of life and quality of dying among residents of nursing homes in Belgium (ultimate impact).

The approach used in this study has led us to the development of an ACP intervention programme that shares some key characteristics with those that have been developed before, such as an emphasis on in-service training for healthcare staff employed by the nursing home [69, 70], providing standardised documentation, conducting structured conversations [69, 71–74] and promoting multidisciplinary awareness [64, 69]. Additionally, important elements were added compared to existing ACP intervention programmes. Firstly, unlike other interventions such as Let Me Talk [74] and the intervention by Morrison et al. in which social workers were trained to perform ACP [48], this intervention programme has a substantial focus on the role of the facility itself. The results of numerous (implementation) projects, including Respecting Choices [58–60, 64, 75, 76], our systematic review [35] and the local experience of stakeholders indicate that a context that supports the implementation of ACP through institutional policy development, management engagement and quality improvement systems is highly valuable [35, 58, 59, 64, 77]. Secondly, our ToC map highlights our hypothesis that a change in desired outcomes through ACP in a setting as complex as nursing homes is hypothesised to be achieved only by targeting multiple levels in a whole-setting approach. Hence ACP cannot be limited to one component (such as training healthcare staff or using a standardised advance directive) but should address multiple levels and domains and take into account a multitude of factors that can inhibit or facilitate its implementation in daily nursing home practice. These factors include high staff turnover (hence the need to continuously train staff), poorly educated staff and the limited number of staff trained in palliative care who are therefore able to recognize signals that it is time to raise subjects relating to ACP.

The main strength of this study is the application of a programme theory via a Theory of Change approach that requires the use of state-of-the-art evidence from research while integrating various stakeholder views in identifying all ToC components, which is different from using a ‘off-the-shelf theory’ such as the Representational Approach to Patient Education to inform the intervention you are developing [33, 78]. The participatory ToC workshops allowed the core research group and stakeholders to discuss in detail the hypothesised preconditions required along the causal pathway and to ensure the initial focus of the ACP intervention always remained on the long-term outcomes that could be achieved with ACP. This contributed to the development of a context-specific ACP intervention whose feasibility is already been partly addressed in the development phase of the study, as recommended by a recent review [42, 79]. Additionally, this study is the first to present a rationale for the particular setup of an ACP intervention programme in nursing homes. It thereby answers a frequent call made by important research bodies to include the rationale, theory or goals that underpin the intervention [26, 28, 79]. Not making explicit how interventions are expected to work makes it challenging for others to replicate and compare existing ACP interventions adequately. It also endangers efforts to scale up and their reliable implementation [30].

This research has several limitations. Firstly, because there is not enough information about the effectiveness of separate components of ACP in scientific literature, the stakeholders and core research group were the main contributors to the development of the overall structure of the ToC map and we were not able to provide high-quality scientific evidence for each link in the causal pathway. Secondly, the number of participants in the workshops was rather small and the heterogeneous composition of each workshop means that lower-level staff may have been less vocal in the discussions due to existing hierarchies. However, we made attempts to mitigate these effects by calling participants without focusing on their profession or rank, and by organising rounds and smaller group discussions. Thirdly, the preconditions identified and the interventions that resulted from our developmental work (situational analysis, systematic review and stakeholder workshops) mainly concern the resident and family level, the staff level, the institutional/organizational level, and the GP collaboration. Other macro level preconditions (defined as “any outside condition or situation that influences the performance of the organization” [77]) such as the regional collaborations with hospitals, the existence of quality indicators or reimbursing providers for ACP conversations, have not been addressed in this work. Finally, the long-term outcomes presented in the ToC map, were chosen in consensus *as the most important long-term outcomes that ACP is directly accountable for* in the context of the Flemish nursing home setting, by the stakeholders involved in our panels and the evidence obtained from the systematic review. As has also been suggested by the EAPC Taskforce on Advance Care Planning, we are aware that there might also be additional outcomes of ACP which future evaluation studies might include [2, 50]. In addition, this visual presentation is of course a simplification of a complex reality. The aim of the ToC approach is to identify the most important and necessary preconditions for implementing ACP successfully, rather than describing every specific element involved. This is hardly feasible, both in practical and financial terms.

Not all results of this study are directly generalizable to other countries. On the one hand, some preconditions are probably also applicable to other countries (i.e. the need for buy-in from management, communication and appropriate monitoring) while some are very specific to the context of Flanders (e.g. using the name ‘reference person’). Our in-depth investigation of the hypothesised process through which ACP can be successfully achieved, can provide researchers in other countries with guidance in developing similar interventions in their country. Within a recent mental health intervention, called PRIME (PRogramme for Improving Mental health carE) [80], the ToC approach proved to be a useful heuristic device for cross-country comparisons and the development and scaling up of mental health services in similar settings. Because the contextual conditions in each country vary significantly and ACP is influenced by a variety of social, political and health system changes, careful documentation and analysis of the context will be essential to interpret future results of ACP evaluations [58].

The results of this study provide the basis for the further design and evaluation of an ACP intervention programme for nursing homes. Developing a ToC is a continual process of reflection and adaptation as barriers to implementation arise and new evidence comes to light. This can require the pathway to be changed and strengthened throughout all phases of the MRC [36]. In the following phase, we will test and possibly further adapt the ToC map and the intervention components in terms of their acceptability and feasibility in the nursing home setting in Flanders. Subsequently, we will evaluate its effectiveness in a cluster randomised controlled trial including an in-depth process evaluation. Because we will develop indicators that will measure the achievement of each precondition, we will be able to gain a detailed understanding of whether the intervention is working, how it works and which components of the complex intervention are the most important in achieving the long-term outcomes. If the intervention does not influence the outcomes as expected, this ToC map will additionally help us to determine whether the lack of effectiveness of the intervention is due to sub-optimal intervention design, implementation failure or genuine ineffectiveness. This is something that past trials have often failed to detect or report [79].

## Conclusion

Within this study, we created a Theory of Change map that describes how and in what circumstances ACP should be implemented and organised in nursing homes to achieve its desired long-term outcomes. We also explicitly state which intervention components should be part of this ACP intervention. The Theory of Change map provides the first comprehensive rationale of how ACP is expected to work in nursing homes, something that has not been shown by research before but for which repeated calls have been made. We will use these insights in the further design of the ACP intervention and its evaluation to explore in greater depth how, why and in what circumstances ACP works best in routine nursing home care in Belgium.

## Additional file

Additional file 1: Script stakeholder workshop 1 and 2. Description of half-day workshops, including timing. (PDF 28 kb)

## Abbreviations

ACP: Advance Care Planning
CAP: Coordinating and Advisory Physician
GP: General Practitioner
ToC: Theory of Change
